# Thyroid Hormones in the Brain and Their Impact in Recovery Mechanisms After Stroke

**DOI:** 10.3389/fneur.2019.01103

**Published:** 2019-10-18

**Authors:** Daniela Talhada, Cecília Reis Alves Santos, Isabel Gonçalves, Karsten Ruscher

**Affiliations:** ^1^Laboratory for Experimental Brain Research, Division of Neurosurgery, Department of Clinical Sciences, Lund University, Lund, Sweden; ^2^CICS-UBI-Health Sciences Research Centre, Faculdade de Ciências da Saúde, Universidade da Beira Interior, Covilha, Portugal; ^3^LUBIN Lab-Lunds Laboratorium för Neurokirurgisk Hjärnskadeforskning, Division of Neurosurgery, Department of Clinical Sciences, Lund University, Lund, Sweden

**Keywords:** brain, recovery, stroke, thyroid hormones, 3,5,3′,5′-tetraiodo-L-thyronine (T_4_), 3,5,3′-triiodo-L-thyronine (T_3_)

## Abstract

Thyroid hormones are of fundamental importance for brain development and essential factors to warrant brain functions throughout life. Their actions are mediated by binding to specific intracellular and membranous receptors regulating genomic and non-genomic mechanisms in neurons and populations of glial cells, respectively. Among others, mechanisms include the regulation of neuronal plasticity processes, stimulation of angiogenesis and neurogenesis as well modulating the dynamics of cytoskeletal elements and intracellular transport processes. These mechanisms overlap with those that have been identified to enhance recovery of lost neurological functions during the first weeks and months after ischemic stroke. Stimulation of thyroid hormone signaling in the postischemic brain might be a promising therapeutic strategy to foster endogenous mechanisms of repair. Several studies have pointed to a significant association between thyroid hormones and outcome after stroke. With this review, we will provide an overview on functions of thyroid hormones in the healthy brain and summarize their mechanisms of action in the developing and adult brain. Also, we compile the major thyroid-modulated molecular pathways in the pathophysiology of ischemic stroke that can enhance recovery, highlighting thyroid hormones as a potential target for therapeutic intervention.

## Introduction

Thyroid hormones (TH), 3,5,3′,5′-tetraiodo-L-thyronine (T_4_) and 3,5,3′-triiodo-L-thyronine (T_3_), are important for brain development in mammals, during embryonic and fetal stages, regulating processes of neuronal proliferation, migration and differentiation, neurite outgrowth, synaptic plasticity, dendritic branching, and myelination (1–5). Also, after birth, TH are crucial for normal brain function throughout the entire life. Specifically for the central nervous system (CNS) the active form T_3_ is a key regulator for normal metabolism in humans and rodents (1, 6, 7).

Availability of T_3_ to the developing and adult brain is tightly controlled by mechanisms regulating TH secretion, free fraction unbound to thyroxine binding globulins (TBG), transmembrane transporters and the activity of iodothyronine deiodinases (DIO). The pattern of these regulatory processes may vary according to the developmental stage and in the adult brain. T_3_ plays an essential role for neurological functions, and minimal disturbances of these mechanisms may have consequences for normal brain development and function (1, 8).

Dependence of the CNS on T_3_ at all stages of development prompted us to review the actions of TH and the relevance of these mechanisms for processes of recovery after ischemic stroke. We will begin to provide an overview on TH signaling in the brain during development and throughout adult life. Thereafter, we will focus on molecules involved in TH signaling after stroke. TH actions at specific time points after the insult, that are dependent of carrier proteins, transmembrane transporters, DIO activity, thyroid hormone receptors (TR) and co-factors, may provide information on underlying molecular and cellular mechanisms that enhance functional recovery of lost neurological functions. Moreover, we will discuss which mechanisms of action of TH in the brain may contribute to enhance functional outcome in stroke patients.

## Thyroid Hormone Transport and Availability to the Human and Rodent Brain

In the adult, TH originate in the thyroid gland that secretes ~93% as T_4_ and 7% as T_3_. Once secreted to plasma, TH binding proteins play an important role to maintain TH homeostasis and distribution into tissues, and <0.1% of TH circulate free in the blood (9, 10). In humans, 65% of TH bind to TBG, 20% to albumin and about 15% to transthyretin (TTR) (11, 12) while in rodents TTR is the main protein carrier in the blood circulation (13, 14). TH also bind to lipoproteins to a less extent (15, 16).

In contrary to processes during brain development, a different fraction of T_3_ in the adult brain is provided from free T_3_ available in the blood circulation and in the cerebrospinal fluid (CSF) (17, 18). Most of TH provided to the brain crosses the blood brain barrier (BBB) and around 20% the blood cerebrospinal fluid barrier (BCSFB) (19, 20). This passage is mediated by transmembrane transporter proteins with overlapping specificity that were identified in endothelial cells of brain microvessels that constitute the BBB and epithelial cells of the choroid plexus (CP) of humans and rodents. These transporters are also important for brain development, and include monocarboxylate transporter (MCT), organic anion transporting polypeptide (OATP), large neutral aminoacid transporter (LAT) and sodium/taurocholate co-transporting polypeptide (SLC10A1) families (17, 21–31). Among those, MCT8 is of particular importance for T_3_ (21, 26, 29), and in mice deficient for this transporter, T_3_ uptake is compromised (32). In humans, MCT10 also facilitates uptake and efflux of TH, in particular for T_3_ (33). The gene that encodes MCT8 (*Slc16a2*) is also present in membranes of neurons, astrocytes, tanycytes and oligodendrocyte precursor cells and OATP1C1 mRNA has been found in astrocytes (34), mediating intracellular TH transport. In addition, it has been proposed that either T_4_ or T_3_ or both are captured via gaps at the endfeet of astrocytes covering brain microvessels (35, 36).

In contrast to the rodent, MCT8 deficiency in humans results in low T_3_ levels in the brain and high levels in the serum due to TH transport deficiency. Thus, the development of the cerebral cortex is impaired accompanied with severe neurological impairment (37–40). The lack of alternative TH transporters such as OATP1C1 (28) and MCT10 (41) in the adult human brain to compensate the transport of T_3_, may contribute to the neurological deficits observed in humans with MCT8 mutations. The activity of DIO2 and DIO3 in the brain is important to balance neuronal intracellular T_3_ levels in the adult brain, according to the developmental stage and brain region (42–44). DIO enzymes catalyze and remove specific iodine atoms from iodothyronine molecules. In rodents, ~50% of T_3_ levels localized in the brain relies on local deiodination of T_4_ in astrocytes and tanycytes by DIO2 (45). T_3_ produced in glial cells is able to promote T_3_ driven transcriptional activity in neurons, demonstrated by *in vitro* experiments in co-cultures of H4 human glioma cells expressing DIO2 and neuroblastoma cells (46). Homeostasis of T_3_ in the CNS is also controlled by DIO3 activity in neurons, that converts T_4_ into 3,3′,5′ reverse triiodo-L-thyronine (rT_3_) and inactivates T_3_ into 3,3′-diiodo-L-thyronine (T_2_) (43, 47, 48).

Other compensatory mechanisms to maintain sufficient T_3_ levels in the rodent brain include the reduction of DIO3 activity and consequently T_3_ degradation, the increase of DIO2 activity in astrocytes (17, 32, 49) and the increase of *Dio2* expression in interneurons in the cerebral cortex (50).

## Thyroid Hormones in Brain Development

It has been shown that mechanisms of brain development might be re-activated in processes of brain reorganization following stroke (51) involving cascades regulated by TH. Therefore, knowledge of TH actions critical and specific for each step of brain development is instrumental to understand their functions following stroke. Epidemiological and clinical studies in humans clearly show that several conditions that compromise maternal TH availability to the fetus impair brain development and are associated with neurological disorders and structural defects, most of them irreversible. A detailed review about TH transport, metabolism and function in the developing brain was recently published (52).

Despite epidemiological and clinical studies have demonstrated the demand of TH during brain development, animal experimental models are of high relevance to identify molecular mechanisms and detailed morphological changes of their biological function during brain development (53). Processes of neurogenesis, proliferation, migration, and maturation show different temporal profiles between humans and rodents, however, basic mechanisms and pathways that regulate brain development are similar (54, 55) allowing the extrapolation of TH deficiency mediated effects in rodents to abnormal TH signaling in humans. Maternal TH are crucial for early cortical neurogenesis, neuronal migration and maturation, during the first trimester of gestation, when fetal brain development occurs (1, 56–58). Both T_4_ and T_3_ are detected in the human brain embryo even before fetal thyroid gland maturation (59) that occurs at 11–12th week of gestation and starts to secrete TH at week 16 (60).

Also in the rat, the embryo is exposed to maternal TH after embryonic day 11 (E11), before the start of thyroid gland development at E17 (61–64). Experimental hypothyroxinemia induced in rats during this period (before E18) causes abnormal neurogenesis in the cortex and hippocampus, leading to impairment of synaptic plasticity and cognitive deficits (65–67), processes of high relevance in mechanisms of recovery after stroke.

Most of TH dependent processes during brain development are due to the interaction of T_3_ with nuclear receptors and regulation of gene expression (68). Increasing levels of protein and mRNA encoding TR alpha and beta (TRα and TRβ) isoforms in the cerebrum and cerebellum start from the 8th to 10th week and increase over gestational time (69, 70) and in rodents there is expression of nuclear TR protein before thyroid gland functioning (71) suggesting transcriptional activity of TH. Several TH dependent genes expressed in the fetal rat brain and neuronal cultures, such as cytoskeletal proteins, are involved in mechanisms of neuronal migration and maturation, branching in neurons and astrocytes (1, 72–74). In both human and rodent species, mutations at the *TR*α and *TR*β result in several neurological disorders (75–79).

The expression pattern of TR in the brain changes during CNS development. TRα1 is the predominant isoform with mRNA and protein expression in the entire brain, in rodents from E14 (80, 81) and in humans from 8th week of gestation (82, 83) onwards, importantly the expression decreases during brain development (84). TRβ1 is expressed at later stages of brain development and in contrary to TRα, TRβ1 mRNA levels do not decrease over gestational time (80, 84, 85). These studies indicate that gene transcription mediated by nuclear TR has spatiotemporal expression patterns and, therefore, TH actions are distinct in all stages of brain development.

In an *in vitro* model of differentiating mouse embryonic stem cell line (ES-E14TG2a) T_3_ treatment (1 nM) enhanced the number of nestin-positive neuronal progenitors, accelerated differentiation and increased survival of pyramidal neurons (86). T_3_ mediated differentiation was associated with the regulation of genes involved in corticogenesis namely *nestin*, empty spiracles homeobox 1 *(Emx1)*, T-box brain gene 1 *(Tbr1)*, Calmodulin kinase 4 *(Camk4)*, and RC3/Neurogranin *(Nrgn)* (86). Regulation of gene expression during differentiation seems to be inversely correlated with levels of chicken ovalbumin upstream-transcription factor 1 (COUP-TF1) (86), that is crucial for adequate neuronal development (87).

## Mechanisms of Thyroid Hormones Actions in the Adult Brain

Cellular actions of TH in the adult brain can be mediated by nuclear receptors and transcriptional activity, and also by non-genomic actions (85, 88, 89). Here we will elaborate in relevant TH actions described in the literature, and below we will delineate TH actions that might be involved in neurorepair processes.

### Genomic Actions of TH

**Figure 1:**
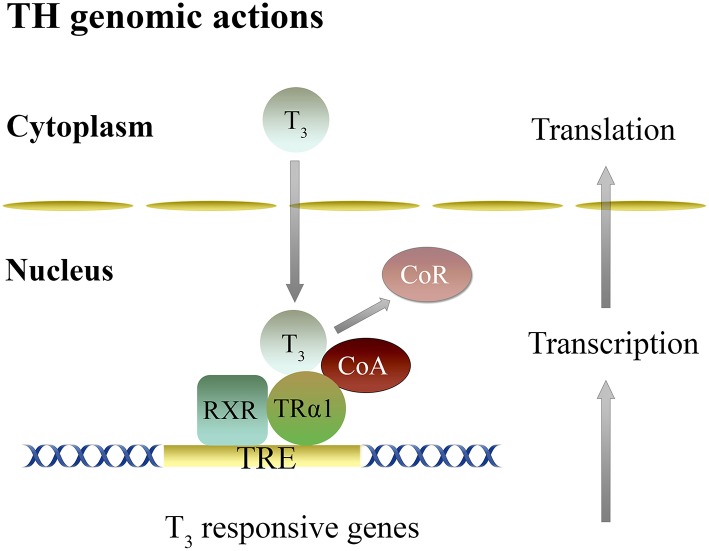
Genomic mechanisms of thyroid hormones action in the brain. Genomic actions of T_3_ are dependent on gene transcription mediated by its binding to nuclear TRα and TRβ, and the formation of heterodimer complex with RXR (RXR-TR) that binds to a TRE, located at the regulatory region of T_3_ target genes. This activity is regulated by an exchange of CoR for CoA. CoA, Co-activator; CoR, Co-repressor; RXR, Retinoid X receptor; TR, Thyroid hormone receptor; TRE, Thyroid response element; T_3_, 3,5,3′-triiodo-L-thyronine.

Actions of T_3_ in the brain are mainly dependent on transcription mediated by T_3_ binding to the nuclear receptors and formation of regulatory complexes (85, 88–91). In the presence of TH, TR are regulated by corepressors (CoR) and coactivators (CoA), proteins, that repress or activate transcription, respectively (36, 85, 88, 89, 92) (Figure 1).

In mammals, there are four isoforms of TR (TRα1, TRα2, TRβ1, and TRβ2) encoded by genes alpha (*Thra*) and beta (*Thrb*), which expression and distribution is different to the developmental brain (93). These isoforms are differently distributed in the tissues, regulate the transcription of different genes and exert different biological actions (94).

TRα1 and TRβ1 are the predominant isoforms in the CNS. TRα1 mRNA and protein accounts to 70–80% of TR expression in the brain (36, 95, 96). Thus, genomic actions of T_3_ in the brain are mainly, but not exclusively, dependent of TRα1 signaling (97). The analysis of brains from TRα1—green fluorescent protein (GFP) mice revealed that this receptor is expressed in all NeuN positive neurons, especially in the nucleus (83). TRα1 is expressed in both excitatory glutamatergic and inhibitory GABAergic neurons in several brain regions including the striatum, cerebral cortex, hippocampus and dentate gyrus, hypothalamus and cerebellum (83, 98). This isoform of TR is also found in tanycytes lining the third ventricle and oligodendrocytes in the hypothalamus, but not in the striatum, somatosensory cortex or hippocampus (83, 99). Its presence in astrocytes is not completely clear (100), however may be dependent on the activation status. Although lower concentration of the receptor is found in cultured rat astrocytes (101), it is not expressed in glial fibrillary acidic protein (GFAP) positive astrocytes in the naïve rat and mouse brain (83, 99). TRα1 is absent in Purkinje cells in the cerebellum (83). Levels of TRα2, a non T_3_ binding isoform, is also detected in the adult brain in a similar pattern as TRα1 (98).

TRβ1 is expressed in the neocortex, and mainly expressed in the pyramidal cell layers of the hippocampus, granule cells of dentate gyrus and paraventricular hypothalamic nucleus (102). It is also expressed in myelin basic protein positive oligodendrocytes (99). In contrary to TRα1, this isoform is highly expressed in Purkinje neurons (94). The TRβ2 isoform is restricted to the anterior pituitary gland and hypothalamus (102–105). As for TRα isoforms, TRβ1 and TRβ2 were not observed in positive GFAP positive astrocytes in the rat brain (99).

Although TR are mainly localized in the cell nucleus and nuclear membrane, TRα1 and TRβ1 isoforms have also been found in the cytoplasm of neurons and astroglia, and this shuttle may increase the rate of T_3_ nuclear import (106). It has been suggested that T_4_ may also exert genomic actions in the brain through binding to TRα1, that is more susceptible to T_4_ than TRβ1 (36), however we did not find experimental studies supporting this hypothesis. So far, a total of 4,108 genes, of which 734 have been identified as being repeatedly regulated by T_3_ in the rodents' brain by microarray analysis (72). In this review, we provide an overview on T_3_-modulated genes that might be involved in brain repair mechanisms (Table 1). Hence, different T_3_–dependent transcriptional activities have been observed in different cell types and brain regions.

**Table 1:** List of genes regulated by thyroid hormones involved in their transport into the brain, mechanisms of tissue repair, and neuronal plasticity following ischemic stroke.

**Genes human/rodent**	**Gene name**	**Function**	**Tissue/cultured cells**	**References**
*SLC16A2/Slc16a2*	MCT8	TH transport	Brain	(107)
*DIO2/Dio2*	DIO2	TH deiodination		
*DIO3/Dio3*	DIO3			
*Bcl2*	Bcl2	Neuronal survival, neurogenesis and neurotrophic factors	Brain cortex	(107–109)
*Vegfa*	VEGFA			
*Sox2*	SRY-box2			
*Ntf*	Neurotrophin			
*Nos2*	NOS2			
*HIF2α*	HIF2α		Neuroblastoma cell line	(107)
*VEGF*	VEGF			
*c-JUN*	c-Jun			
*ENO2*	Enolase-2			
*Emx1*	empty spiracles homeobox 1		mES cell line	(86)
*Tbr1*	T-box brain gene 1			
*Bdnf*	Brain derived neurotrophic factor		Hippocampal slices	(110)
*Slc12a5*	KCC2			
*NRGN/Nrgn*	Neurogranin	Synaptic plasticity	Hippocampus and forebrain/mES cell line	(86, 111–113)
*CAMK4/Camk4*	Calmodulin kinase 4		Brain/Neurons/mES cell line	(86, 114–117)
*Reln*	Reelin		Brain	(118–120)
*Srg1*	Synaptotagmin-related gene 1		Brain	(121)
*Nefh*	Neurofilament heavy polypeptide		Neurons	(122)
*Nefm*	Neurofilament medium polypeptide			(122)
*GFAP*	GFAP		Astrocytes	(123)
*Vim*	Vimentin		Mesenchimal cells	(122)
*Nes*	Nestin		Neurons	(86, 122)
*Vegf*	VEGF	Angiogenesis	Brain	(124–126)
*Angpt2*	Angiopoietin-2			

### Non-genomic Actions of TH

TH non-genomic actions that do not require TH binding to nuclear receptors are well-described in the literature (127–130). Actions are immediate and include several interactions of TH with extranuclear receptors, including TRα and TRβ, located in the cytoplasm, cellular membrane, cytoskeleton and mitochondria, modulating several intracellular pathways.

**Figure 2:**
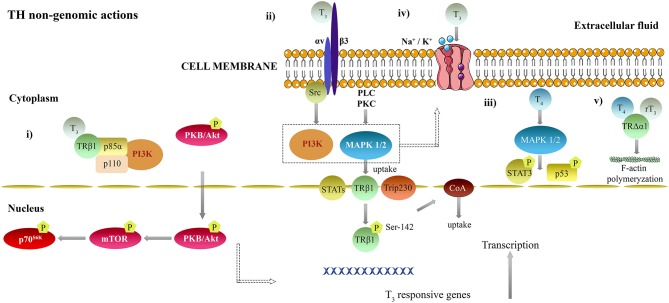
Non-genomic mechanisms of thyroid hormones action in the brain. T_3_ can also modulate other genes not containing TRE, by non-genomic actions. **(i)** T_3_ complexed to TRβ1 in the cytoplasm interacts with p85α subunit of PI3K, resulting in phosphorylation and activation of PKB/Akt signal transduction pathway, mTOR and phosphorylation of p70^S6K^ (131–136). **(ii)** T_3_ is able to bind to integrin αvβ3 S1 domain in plasma membranes and activates PI3K via Src kinase. T_4_ and T_3_ interact with integrin αvβ3 S2 domain and activate MAPK 1/2 signaling cascade, PLC and PKC (127, 133, 137–140). Subsequently, it results in an nuclear translocation of TRβ1 (141), estrogen receptor α (142), STAT1α, IFN-γ (143) and CoA protein Trip230 (144). In addition, hormone activated MAPK 1/2 phosphorylates TRβ1 at Ser-142, leading to recruitment of CoA proteins (145). **(iii)** T_4_ non-gnomically activates MAPK 1/2 in HeLa and CV-1 cultured cells (146, 147) and phosphorylation of p53 (148) and STAT3 (147). **(iv)** T_3_ modulates Na^+^/H^+^ exchanger in myoblasts (149); Na-K-ATPase activity in alveolar epithelial cells (150–152), embryonal hepatocytes (153) and synaptosomes (154, 155) through either the PI3K or MAPK pathways (152, 156) the Ca-ATPase activity in erythrocytes (88), the sarcoplasmic reticulum in the heart (157) and in cerebrocortical synaptosomes (158). **(v)** T_4_ and rT_3_ stimulate polymerization of actin components of the cytoskeleton neuronal and astrocyte cell cultures, through TH binding to an extranuclear truncated form of TRα1 (TRΔα1) (159–162). IFN-γ, interferon gamma; MAPK 1/2, mitogen-activated protein kinase 1/2; PLC, phospholipase C; PI3K, phosphatidylinositol 3-kinase; PK - protein kinase; mTOR – rapamycin; STAT - signal transducing and activator of transcription; TH, Thyroid hormones; TR, Thyroid hormone receptor; TRE, Thyroid response element; T_4_, 3,5,3′,5′-tetraiodo-L-thyronine; T_3_, 3,5,3′-triiodo-L-thyronine.

The following points summarize relevant non-genomic actions of TH binding to membranous and cytoplasmic receptors (i–iii), cytoplasmic TH binding proteins affecting ion pumps activity (iv) and the action of TH on the cytoskeleton (v) (Figure 2). (i) T_3_ complexed to TRβ1 in the cytoplasm interacts with p85α subunit of phosphatidylinositol 3-kinase (PI3K), resulting in phosphorylation and activation of protein kinase (PK) B/Akt signal transduction pathway, rapamycin (mTOR) and phosphorylation of p70^S6K^ (131–136). (ii) T_3_ is able to bind to integrin αvβ3 S1 domain in plasma membranes and activates PI3K via Src kinase. T_4_ and T_3_ interact with integrin αvβ3 S2 domain and activate mitogen-activated protein kinase 1/2 (MAPK 1/2) signaling cascade, through phospholipase C (PLC) and PKC (127, 133, 137–140). Subsequently, it results in an nuclear translocation of TRβ1 (141), estrogen receptor α (142), signal transducing and activator of transcription (STAT) 1α, interferon gamma (IFN-γ) (143) and CoA protein Trip230 (144). In addition, hormone activated MAPK 1/2 phosphorylates TRβ1 at Ser-142, leading to recruitment of CoA proteins (145). (iii) T_4_ non-gnomically activates MAPK 1/2 in HeLa and CV-1 cultured cells (146, 147) and phosphorylation of p53 (148) and STAT3 (147). (iv) T_3_ modulates Na^+^/H^+^ exchanger in myoblasts (149); Na-K-ATPase activity in alveolar epithelial cells (150–152), embryonal hepatocytes (153), and synaptosomes (154, 155) through either the PI3K or MAPK pathways (152, 156); the Ca-ATPase activity in erythrocytes (88), the sarcoplasmic reticulum in the heart (157) and in cerebrocortical synaptosomes (158). (v) T_4_ and rT_3_ stimulate polymerization of actin components of the cytoskeleton neuronal and astrocyte cell cultures, through TH binding to an extranuclear truncated form of TRα1 (TRΔα1) (159–162).

## Thyroid Hormones in the Aging Brain and Ischemic Stroke

The complex process of aging is associated with changes in TH metabolism and action in all tissues. During aging, the disruption of circadian rhythm leads to a reduction in thyroid stimulating hormone (TSH) secretion (163–165) and circulating TH levels, in particular T_3_, in humans (166, 167) and rodents (168). Nevertheless, TH signaling is well-preserved in the aging brain, as demonstrated in mouse models of aging (169). However, hypothyroidism and decreased TH availability to the brain has been considered a risk factor for the development of neurodegenerative diseases (170, 171) and acute ischemic stroke (172, 173). In addition, recent epidemiological studies have associated low levels of T_3_ with poor functional outcome after acute ischemic stroke (174–179). Interestingly, lower total T_3_ levels is not related with poor functional recovery after ischemic stroke in patients below 65 years of age, suggesting that the association between levels of T_3_ and stroke recovery may be clinically important in older patients (180). Non-thyroidal illness syndrome also impairs functional recovery after stroke (181). Besides, stroke patients with thyroid dysfunction (lower levels of TSH and higher levels of free T_4_) are associated with poorer clinical outcome (182). Together, studies point toward the need for a systemic assessment of thyroid dysfunction and stroke outcome.

Some reports suggest neuroprotective effects of hypothyroidism prior to brain ischemia in humans (183, 184), as well as experimental studies in rodents (185, 186). In a recent systematic review, stroke patients with subclinical hypothyroidism (higher levels of TSH and normal levels of free T_4_ within the reference range) were more prone to suffer a non-fatal stroke and minor adverse events (182). However, we lack mechanistic studies how systemic levels of TH exactly influence processes in the postischemic brain. It is likely that hypothyroid episodes prior to stroke only delayed neuronal death, due to decreased metabolic demand of neurons, decreased glutamate production and delayed oxidative stress (185, 187). There is no evidence from experimental studies that show beneficial effects in hypothyroid animals after stroke. A recent animal study suggested that daily intravenous administrations of rT_3_, an inactive form of T_3_, prevents ischemic-reperfusion injury in rats subjected to transient MCAO, however authors did not evaluate if rT_3_ induced an hypothyroid state (188). Similarly, in rats subjected to permanent middle cerebral artery occlusion (MCAO), TH serum levels are reduced 14 days after injury correlated with increased neurological impairment (189).

On the other hand, hyperthyroidism has been associated with an increased risk for ischemic stroke in humans (190–192). However, the population-based study was performed in patients aged 18–44 years. Hence, this study has not been adjusted for other risk factors such as hypertension and atrial fibrillation that may independently contribute for stroke. Larger infarct volumes also have been found in hyperthyroid rats after transient MCAO (193). Hence, hyperthyroid rats (oral administration of TH for 4 weeks) showed profound effects on the cardiovascular system including hypertension and tachyarrhythmia and treatment resulted in a catabolic metabolism (193).

Interestingly, increased mRNA expression of *Dio2* has been found in astrocytes during the first 72 h after transient MCAO (194). Together with modulation of *Thrb* expression, that is reduced in the infarct core and increased in the peri-infarct area, it suggests a local action of T_3_ (189). Repeated daily administrations of T_4_, before and on days one, two and three after stroke, decrease neuronal damage in the cornu ammonis CA1 pyramidal cells in the hippocampus (195). In an animal model of MCAO, intraperitoneal injection of T_4_ (11 μg/kg, 1 h after ischemia and 6 h after reperfusion) reduced cortical and striatal infarct volume 24 h after stroke, with a reduction of GFAP, Iba-1, PKC, and MAPK 1/2 expression (196). Treatment with levothyroxine (25 μg/kg intraperitoneal) 1 h after traumatic brain injury stimulated mRNA expression of genes encoding MCT8, DIO2, and DIO3; genes related with neuronal survival and neurogenesis, namely *Bcl2*, vascular endothelial growth factor A (*Vegfa*), *Sox2*, and neurotrophin (*Ntf* ) in the cortex, and of inducible nitrite oxide synthase 2 (*Nos2*) (107).

Moreover, intraperitoneal administration of T_3_ at 12 μg/kg 1 h after traumatic brain injury reduced lesion size and inflammation (197). T_3_ treatment 25 μg/kg 30 min after transient MCAO also reduced volume of stroke damage in mice through stimulation of fatty acid oxidation by astrocytes (198). A combination therapy of bone marrow stromal cells, daily injections of T_3_ 200 μg/kg and mild exercise was related to reduce ischemic damage 7 days after transient MCAO in rats (199). Likewise, intraperitoneal administration of thyroxine derivates, 3-iodothyronamine and thyronamine, 50 mg/kg 1 h after MCAO in mice, also reduced infarct volume (200). Neuroprotective action of 3-iodothyronamine administered 2 days before MCAO was associated with hypothermia (200). Although molecular mechanisms have not been evaluated, these studies suggest that non-genomic actions of TH contribute to neuroprotection in the acute phase following stroke. In addition, T_3_ treatment prior to brain ischemia (25 μg/kg intravenous) has prevented edema through suppression of aquaporin-4 (AQP4) water channel expression and thereby reducing infarct volume and improving neurological outcome after transient MCAO (109), effects that were enhanced when T_3_ was administrated in nanoparticles at equivalent doses (108). Recently, it has been demonstrated that T_3_ modulates AQP4 expression dependent on developmental stage of the CNS. Treatment of mice with T_3_ at 1 μg/g increased AQP4 expression in astrocytes in the cerebral cortex until the 60th postnatal day. In contrast, whereas in glioblastoma cell lines stimulation with T_3_ (50 nM) treatment was downregulating the expression of AQP4 (201).

Few experimental studies have been performed to identify the mechanisms of TH actions in neuroprotection. Treatment of mouse hippocampal slices lesioned between CA3 and CA1 with T_4_ increases levels K-Cl cotransporter (KCC2) mRNA in a brain derived neurotrophic factor (BDNF) dependent manner, that promoted survival and regeneration of damaged neurons in the CA1 region (110). Moreover, it has been demonstrated that treatment with T_3_ has a protective effect against glutamate toxicity in cultured astrocytes and neurons (202, 203). This action has been linked to non-genomic actions of TH on Na^+^/H^+^ exchange activity and glutamate transport (204). Also, T_3_ treatment stimulated the expression of *HIF2*α, *VEGF, c-JUN*, and Enolase-2 (*ENO*2) in the neuroblastoma in an *in vitro* hypoxia model (107). Although only a few scattered studies have been performed, they indicate an involvement of T_3_ in pathways that promote neuronal protection and recovery, through genomic or non-genomic actions.

The expression of TR in the human brain after ischemic stroke have not been studied. Also in experimental models, TR expression has not been investigated, and we found only one experimental study reporting TR expression after permanent MCAO (189). Interestingly, TRα1 expression is increased in microglial cells in the infarct core and in neurons in the peri-infarct area. Astrocytes mildly express nuclear TRα1 and expression of TRβ1 is strongly expressed in the astrocytic scar. If TRα1 and TRβ1 play a crucial role for recovery after brain stroke, in humans and rodents, it remains to be investigated. In fact, TRα1 have been demonstrated to play a crucial role for cardiomyocyte survival after myocardial infarction (205) Therefore, the idea that TRα1 could be a target to promote stroke recovery definitively needs to be further investigated.

## Mechanisms of Thyroid Hormones that may Enhance Mechanisms of Recovery After Stroke

Beyond the acute phase after stroke, the brain shows the capacity of spontaneous recovery of lost neurological functions albeit to a limited extent. This process remains slow, however, the intrinsic mechanisms are present and patients may benefit from enhancing those. TH regulate several pathways that are involved in neurorepair, namely regulation of processes of neuronal plasticity, neurogenesis, angiogenesis, and glutamate toxicity. Adjuvant therapies that modulate those processes may improve recovery of function after stroke, in particular when applied in combination with physiotherapy in stroke patients or an enriched environment in rodent models of stroke (206, 207).

### Neuronal Plasticity

Neuronal reorganization occurs during the recovery phase of stroke and is initiated by cellular responses to degeneration. Cell death in the infarct core results in synaptic degeneration, instigating regenerative responses among surviving neurons, as the formation of new synaptic connections (208). Neuronal plasticity includes all mechanisms involved in modulation of dendritic and axonal arborization, dendritic spine density and neuronal density that will determine the formation of new synaptic connections and neuronal networks (209, 210).

Neuroplasticity occurs spontaneously during stroke recovery and TH have been identified as a modulator of several genes that may stimulate endogenous neuroplasticity and therefore contributing to facilitate recovery (Table 1).

In rodents, T_3_ regulates neuron specific RC3/Neurogranin gene (*Nrgn*), that encodes a calmodulin binding protein (112) which binds to calmodulin in the absence of calcium distribution in spines and enhances synaptic plasticity (211). *Nrgn* is highly expressed in dendritic spines in the hippocampus and forebrain and deficiency of *Nrgn* in mice has been reported to induce deficits in spatial learning and anxiety-like tendencies (113). In the human, the homolog gene NRGN is also a direct TH target, during development and in the adult brain (111). TH also regulates *Reelin* (*Reln*) expression during brain development (118). Administration of T_3_ increases *Reln* expression in the hippocampus of adult rats (212). Reelin is involved in the migration of multipolar neurons in the developing neocortex (120) and in the adult brain interacts with apoliprotein E receptors and regulates synaptic plasticity and neurogenesis (119).

During brain development, T_3_ regulates genes related with the calcium/Calmodulin-activated kinase 4—cAMP responsive element-binding protein 1 (CaMK4/Creb1) signaling pathway, as demonstrated in cultured fetal neurons (213), a mouse embryonic stem cell line (86) and *in vivo* studies (114–117). The CaMK4/Creb1 pathway regulates calcium influx and dendritic growth during development (214), inhibits apoptosis through phosphorylation of Creb and increases anti-apoptotic gene expression. Synaptotagmin-related gene 1 (*Srg1*) is also a TH responsive gene during brain development, that has a putative role as a mediator of synaptic structure and activity (121).

Reorganization of spine cytoskeleton, principally microtubules and actin filaments, can be dynamically modulated and consequently change the pattern of synaptic activity (215). Several studies have shown that TH modulate tubulins (216, 217), microtubule associated proteins (218) and Tau protein (219) in the cytoskeleton during brain development and in the adult brain. TH has also been demonstrated to modulate transcription of genes of intermediate filaments, namely neurofilaments (*Nefh* and *Nefm*), GFAP in mature astrocytes, vimentin in mesenchymal cells and nestin (116, 122). Experiments in cerebral cortex slices suggest that TH activates phosphorylation of cytoskeletal proteins mediated by GABAergic signaling dependent on PKA and PKCaMII activity (220). Studies conducted in cultured glial cells also suggests that TH reorganize the cytoskeleton through GFAP phosphorylation mediated by RhoA signaling pathway (221).

Actions of TH in the cytoskeleton are particularly important during brain development, to guarantee proper cell migration and to foster neurite outgrowth (160). T_3_ also regulates transcription of genes involved in cytoskeleton formation in neurons and astrocytes, during fetal and postnatal brain development (74). Hypothyroidism leads to impaired actin cytoskeleton formation in neurons and astrocytes, affecting neuronal migration and neurite outgrowth. Both rT_3_ or T_4_ administration can restore polymerization of intracellular filaments F-actin (222, 223) and laminin (223, 224), but this effect was not observed by T_3_ administration. Also in neuronal and astrocyte cultures, T_4_ and rT_3_ stimulate polymerization of the actin cytoskeleton, as already mentioned above (Figure 2) (159, 160, 162). As during brain development, basic transcription element-binding protein is upregulated by TH and this protein may play a role in neuronal outgrowth, modulating changes in the cytoskeleton, and cell differentiation (225, 226).

Studies also demonstrate that TH signaling is critical to proper functioning of short term (227) and long term synaptic plasticity (228). Induced hypothyroidism has been related with disruption in synaptic plasticity (229, 230) and long term potentiation in the CA1 neonatal (231, 232) and adult (233) rat hippocampus. Hyperthyroidism has been also related to detrimental effects in dendritic spines. Intraperitoneal injection of T_3_ 750 μg/kg during 5 consecutive days in adult rats significantly decreased dendritic spine density in CA1 pyramidal cells in the hippocampus (234) and thyroxine induced hyperthyroidism impairs special learning and synaptic plasticity in rats (235).

On the cellular level, TH genomic or non-genomic actions may modulate the activity of ion pumps that are important for normal excitable cell function. Particularly in brain tissues affected by ischemia, directly or indirectly, adapted function of ion pumps is required to avoid intracellular overload of H^+^ and Ca^2+^, preventing cell acidosis and excitotoxicity. T_3_ has been shown to decrease the activity of Na, K-ATPase (154, 155) and to stimulate the Na^+^/H^+^ exchanger (149) and Ca^2+^/Mg^2+^ ATPase pump activity (158) in cerebrocortical synaptosomes. T_3_ increases the transcription of SR Ca^2+^-ATPase gene (ATP2A2) in the sarcoplasmic reticulum (157). In addition, T_3_ has been demonstrated to be benefical in *in vitro* and *in vivo* experimental myocardial ischemia preventing excessive intracellular Ca^2+^ accumulation (236, 237).

Also, T3 contributes to glutamate uptake by astrocytes, protecting neurons from intracellular calcium toxicity and death (203). Neuroprotective effect was attributed to an increased expression of mRNA and protein levels of GLT-1 and GLAST in the astrocytes. It also has been demonstrated that T3 decreases N-methyl-d-aspartate (NMDA)-evoked currents and prevent glutamate-induced neuronal death in hippocampal neurons (202). Together, these actions might be beneficial to prevent cell dysfunction or death of principal neurons during the acute phase after ischemic stroke. Conversely, these mechanisms might be involved to reduce the activity of inhibitory neurons in critical periods of plasticity during the first weeks after stroke. Hence, these actions most likely will dependent on receptor expression profiles in different neuron populations.

The balance between excitation and inhibition is of particular importance for neuronal plasticity processes potentially relevant for recovery. During development, TH increases the level of γ-aminobutyric acid (GABA) in the brain, while the opposite effect is observed in the adult brain. In the developing brain, hypothyroidism impairs the generation of interneurons including reduced proliferation and delayed differentiation of precursor cells in the cerebellum and their migration to the cerebellar cortex (238). These effects could be antagonized by administration of T_3_ binding to the TRα1. Likewise, deletion of TRα1 reduced cerebellar GAT-1 expression and Pax-2 precursor cell proliferation (238). TH also affect the release and uptake of GABA from the neuron into the synapse. T_3_ stimulates depolarization and release of GABA in synaptosomes from rat cerebral cortex (239). In the adult brain, hypothyroidism is reported to increase glutamic acid decarboxylase (GAD) activity and GABA reuptake, from cerebral cortex homogenates (240, 241) while hyperthyroidism has no effect on GABA uptake (241). In addition, T_3_ administration inhibits GABA-induced Cl^−^ currents, which may affect GABA_A_ receptors in the cerebral cortex, by non-genomic mechanisms (242).

### Adult Neurogenesis

TH signaling is crucial for proper neurogenesis during brain development (1). Several studies have demonstrated that neurogenic events in the adult brain are dependent on TH actions (243–250) and have been reviewed in detail (251, 252). Particularly T_3_ is involved in mechanisms of proliferation, survival, differentiation and maturation of neuronal precursors in the adult brain (246, 251). With potential contribution of TH NSPCs from the SVZ may proliferate, migrate and differentiate into neurons, astrocytes or oligodendrocytes in the damaged region and thereby contribute to brain plasticity after ischemic stroke or other brain injury (253, 254). Stem cell therapy and neurogenesis have been explored as a potential therapeutic strategy for neuronal repair after ischemic stroke (255).

### Angiogenesis

Therapeutic angiogenesis has been used to enhance brain repair promoting the formation of new blood vessels and restoration of blood flow in the damaged area (256–258). Angiogenic effects of TH have been demonstrated in infarcted tissue of the myocardium (259). Moreover, an increased number of new blood vessels has been found in the brain of hypothyroid rats after administration of 3,5-diiothyroprionic acid (a thyroid hormone analog) or T_4_ (260). The proangiogenic effects of TH are apparently mediated by non-genomic actions, through binding to integrin αvβ3 resulting in activation of MAPK 1/2 and STAT3. TH binding to αvβ3 directs transcription of genes that promote angiogenesis, namely fibroblast factors, VEGF and angiopoietin-2 (124–126, 137, 147, 261, 262). To our knowledge, so far no experimental studies have been performed to investigate pro-angiogenic effects of TH after stroke.

## Translation to Clinical Studies

Current epidemiological studies in humans and experimental evidence from rodents strongly suggest that TH signaling plays a crucial for stroke recovery. In particular T_3_, the active form in the brain, exerts genomic and non-genomic actions that may foster functional outcome after stroke.

Although several epidemiological studies have associated low levels of TH with poor outcome, no clinical trials have been performed to evaluate the recovery promoting effects of TH in stroke patients. At the current stage the first step of translational studies will be to understand exact mechanisms underlying beneficial action of TH after stroke in animal models, in particular T_3_. Based on knowledge about mechanisms of action, exact treatment regimens with specific targets can be developed and tested during critical windows of stroke recovery. In this context, the development of cell-specific approaches to target TH signaling in the postischemic brain may result in specific treatments in experimental stroke models, that later, might be translated into clinical studies.

## Conclusions

Several mechanisms in the brain are tightly regulated by TH and T_3_ availability to the brain is dependent on factors including (i) maternal TH release before fetus thyroid gland development; (ii) TSH levels and TH release by thyroid gland; (iii) passage of TH through placenta in the fetus; (iv) control of free fraction of TH determined by TH binding proteins; (v) TH transmembrane transport into the cytoplasm; (vi) local activity of iodothyronine deiodinases; (vii) expression and distribution of TR; (viii) and translational activity and non-genomic actions of TH in the brain. Disruption in these mechanisms compromise TH availability and actions in the brain and may result in impairments of neurological functions. There is clinical and preclinical evidence that TH are involved in mechanisms of neuronal plasticity and function of glial cells after ischemic stroke. Further understanding and targeting those might be exploited in future therapies to enhance functional recovery in stroke patients.

## Author Contributions

DT conceptualized and wrote the review, under supervision of KR. KR, CS, and IG revised the manuscript. All authors approved the final version of the manuscript for submission and publication.

### Conflict of Interest

The authors declare that the research was conducted in the absence of any commercial or financial relationships that could be construed as a potential conflict of interest.
